# Mouse Vocal Fold Permeability In Vivo: Effects of Novel Low‐Tech Injury and Instillation Methods

**DOI:** 10.1002/lary.70266

**Published:** 2025-12-10

**Authors:** Renee E. King, Ella T. Ward‐Shaw, Paul F. Lambert

**Affiliations:** ^1^ McArdle Laboratory for Cancer Research, Department of Oncology University of Wisconsin‐Madison Madison Wisconsin USA

**Keywords:** animal model, drug delivery, epithelial permeability, larynx, vocal fold injury

## Abstract

**Objectives:**

Vocal fold (VF) injury and instillation are emerging techniques to study laryngeal pathologies in mouse models. Most approaches require high‐tech visualization and lengthy anesthesia. Intraperitoneal (IP) naphthalene (NAPH) and topical polidocanol (PDOC) are two chemical methods of injuring murine lower airways. Oropharyngeal aspiration (OA) is used for murine lung instillation. We assessed whether these simple low‐tech methods could injure and instill mouse VFs, and whether injury increased in vivo VF epithelial permeability.

**Methods:**

Mouse VFs were injured using IP NAPH at 200, 250, 300, or 350 mg/kg, or OA of PDOC at 0.5% or 2% w/v. Twenty‐four hours later, mice received Evans blue (EB) dye OA instillation, then were sacrificed after 30 min. Coronal larynx sections were assessed for VF injury. Permeability was measured by imaging EB autofluorescence and quantifying percent positive area and signal intensity. VFs were immunostained for basal cells (p63), tight junctions (ZO‐1), and basement membrane (laminin).

**Results:**

VF injury was 100% with 350 mg/kg NAPH or high‐volume 2% PDOC and 0%–40% with other treatments. EB bound VF lamina propria in up to 100% of mice in a volume‐dependent manner. Permeability did not differ by injury. Basal cells and tight junctions were decreased in injured VFs. Basement membrane was largely intact.

**Conclusions:**

High‐dose NAPH consistently injures mouse VFs. OA reliably instills mouse VFs. Uninjured murine VF epithelium is highly permeable to low molecular weight dye in vivo. Inherent permeability of mouse VFs may facilitate targeted genetic engineering approaches and studies of environmental hazards and drug treatments.

**Level of Evidence:**

n/a.

## Graphical abstract

Improving experimental mouse vocal fold injury and instillation approaches is necessary to advance preclinical models of laryngeal disease. We have successfully repurposed low‐tech, quick, and reliable injury and instillation methods established in respiratory research. Using these methods, we show that murine vocal fold epithelium is inherently highly permeable, and that permeability does not increase with injury.

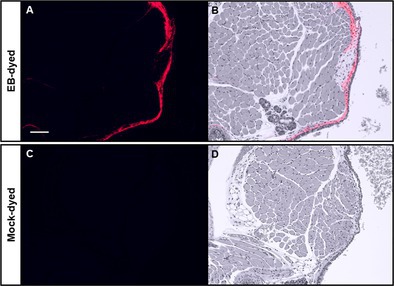

## 1 Introduction

Mouse models of voice disorders are a growing area of laryngology research. Experimental mouse vocal fold (VF) injury is necessary to generate models that are relevant to human voice disorders. Surgical methods for murine VF injury include cutting, scratching, and abrading mucosa with a needle or brush [1, 2, 3, 4, 5, 6, 7]. Surgical injury is performed under endoscopic and/or microscopic visualization. High‐tech visualization provides real‐time procedure verification. However, there are disadvantages. Endoscopy and microscopy equipment is expensive, bulky, and fragile, which poses challenges for transportation to animal facilities, placement in small procedure rooms and biosafety cabinets, and sanitization and sterilization of endoscopy equipment. Surgical VF procedures require injected anesthetics or continuous delivery of isoflurane gas with access to the oral cavity. For some experiments, multiple investigators are needed to simultaneously operate visualization equipment, perform injury, and monitor anesthesia. Procedures are technically challenging and have a steep learning curve. These factors increase complexity and cost, decrease reliability and reproducibility, and increase risks of morbidity and mortality.

Chemical injury methods hold promise for simplifying and improving the reliability of murine VF procedures. Naphthalene (NAPH) and polidocanol (PDOC) are two chemicals commonly used in mice to injure respiratory epithelium [8, 9, 10, 11, 12, 13, 14, 15, 16, 17, 18]. NAPH is an aromatic hydrocarbon found in coal tar and mothballs. NAPH delivered by intraperitoneal (IP) injection was first established for experimental murine lung injury in the 1970s [19, 20]. NAPH selectively ablates club cells, which comprise 9% of the airway epithelium and are not found elsewhere in the body [21]. In the mouse larynx, 275 mg/kg IP NAPH does not grossly injure VFs on day 1 but ablates VF club cells and induces hyperplasia by day 3 [22]. PDOC (C_30_H_62_O_10_, aka nonaethylene glycol monododecyl ether [23]) is a surfactant and sclerosing agent used to treat varicose veins. Case reports describe intralesional PDOC injection for vascular laryngeal and pharyngeal lesions [24, 25]. When applied topically to murine airway epithelium, 1%–2% PDOC can either denude the epithelium or remove terminally differentiated cells while leaving basal cells intact [8, 9, 26]. Topical PDOC increases the permeability of nasal, tracheal, and pulmonary epithelium to drugs, viral vectors, and cells [26, 27, 28]. To our knowledge, topical PDOC has not been tested in VFs. We hypothesized that both NAPH and PDOC could be calibrated to reliably injure VFs.

Mouse models of laryngeal disease require exposing VFs to reagents, pathogens, environmental hazards, and experimental drugs. While some substances can be delivered to murine VFs by inhalation [29, 30] or the natural flow of aerodigestive secretions [31, 32, 33], direct instillation could potentially deliver any liquid solution. Instillation has been used to apply gastric reflux components [3], papillomavirus [5], and chemical carcinogens [4] to murine VFs. However, these procedures were performed under high‐tech visualization, which carries the risks discussed above. In contrast, oropharyngeal aspiration (OA) is a low‐tech method that is well‐established for mouse lung instillation [34]. OA involves isoflurane anesthesia and quickly pipetting liquid into the airway via the oral‐tracheal route.

The effect of injury on VF epithelial permeability has yet to be fully explored. Papillomavirus can enter uninjured laryngeal epithelium and cause disease in immunodeficient mice [5, 32]. Transepithelial electrical resistance (TEER), which is inversely associated with permeability, is lower in murine larynx than esophagus [35]. Therefore, murine laryngeal epithelium may be inherently permeable. However, there is evidence that injury increases murine laryngeal epithelial permeability. Laryngeal abrasion hastens papillomavirus disease incidence and increases severity [5]. Exposure to gastric reflux decreases TEER and increases permeability to fluorescein ex vivo [31]. Murine VF permeability has not been systematically assessed in vivo.

The goals of this study were to assess the extent to which NAPH and PDOC injure murine VFs, to determine whether OA reliably instills murine VFs, and to test the effect of VF injury on in vivo epithelial permeability. To answer these questions, we used varying concentrations of NAPH and PDOC to injure VFs and assessed the permeability of injured and mock‐injured VFs to tracking dye (Experiment 1). Subsequently, we tested the effect of higher dye OA volumes on the reliability of VF instillation (Experiment 2), then assessed the effects of higher PDOC OA volumes on VF injury and permeability (Experiment 3).

## 2 Materials and Methods

### 2.1 Animals

A total of 96 male and female FVB/N mice (Taconic Biosciences) aged 6–13 weeks old were used in three experiments, described in the Study Design section below and Table S1. Animals were allocated to obtain quantitative measurements of permeability from at least four mice per dyed group, and to balance sex distribution in each experiment. More mice than this minimum number were included in some groups due to variable litter sizes. Then, additional animals were added to provide mock‐dyed control tissues. Procedures were approved by the University of Wisconsin‐Madison Institutional Animal Care and Use Committee (IACUC #M005871).

### 2.2 VF Injury

Animals underwent VF injury via NAPH IP injection or PDOC VF instillation. NAPH (Sigma‐Aldrich PHR1275) was dissolved in corn oil at 20–35 mg/mL. Mice were injected IP with 10 μL/g body weight for a final dose of 200–350 mg/kg. PDOC (Sigma‐Aldrich P9641) was dissolved in phosphate‐buffered saline (PBS) at 0.5% or 2% w/v. VFs were instilled with 10 or 25 μL PDOC. Mock‐injured mice were instilled with 10 or 25 μL PBS.

VF instillation was performed using OA. Anesthesia was induced with 3% isoflurane. Mice were removed from the anesthesia chamber and suspended by the maxillary incisors from a loop of suture attached to a four‐way tube rack (Heathrow Scientific) placed vertically and secured at a ~75° angle. The tongue was removed from the mouth using forceps and held between thumb and forefinger. Solution was pipetted into the oral cavity. The tongue was restrained to prevent swallowing and the nares were covered with a fingertip until the mouse took 3–5 deep breaths through the oral cavity, thereby aspirating the solution. Aspiration was confirmed by listening for initiation, then cessation of wet‐sounding breaths, and visual inspection for liquid in the oropharynx. Mice were placed prone until anesthesia recovery.

### 2.3 VF Dye Instillation

Evans blue dye (EB; 961 Da; Sigma‐Aldrich E2129) was dissolved in PBS at 5% w/v. EB was selected because it autofluoresces bright red [36] and strongly binds collagen and elastin [37], which are abundant in VF lamina propria. Mice were instilled with 10–50 μL EB using OA as described above. Mock‐dyed mice were instilled with 10–50 μL PBS.

### 2.4 Study Design

For each experiment, all animals were treated on the same day or days with the same aliquot of reagents.

#### 2.4.1 Experiment 1: VF Injury and Permeability

To test whether VF injury increased permeability, we injured VFs and instilled EB dye 24 h after injury. Injury treatments were 200, 250, 300, and 350 mg/kg IP NAPH; 0.5% and 2% PDOC OA; and PBS OA mock injury (*n* = 4–7 per group; Table S1). PDOC and dye were instilled in 10‐μL volumes based on preliminary experiments (Figure S1). For mock‐injured, NAPH 350, and 2% PDOC groups, two mice per group were mock‐dyed with PBS.

#### 2.4.2 Experiment 2: High‐Volume Dye

To test whether higher OA volumes would improve VF instillation, we instilled 25 or 50 μL EB (*n* = 16 per group; Table S1) or PBS mock dye (*n* = 3 per group).

#### 2.4.3 Experiment 3: High‐Volume PDOC

To test whether high‐volume PDOC would increase VF injury, we instilled 25 μL of 0.5% PDOC, 2% PDOC, or PBS mock injury (*n* = 5–8 per group; Table S1) and instilled EB dye 24 h later to assess permeability. Two mock‐injured mice and two mice treated with 2% PDOC were mock‐dyed.

### 2.5 Histology

All animals were sacrificed and tissues were collected 30 min after dye instillation. Larynx, trachea, lungs, and esophagus were assessed for the presence of blue dye. Tissues were then fixed in 10% formalin for 24 h, processed, and embedded in paraffin. Five micrometer coronal larynx sections were cut. Every 10th slide was stained with hematoxylin and eosin (H&E). VFs were viewed with a Zeiss AxioImager M2 microscope running AxioVision software and assessed for injury, defined as the presence or absence of epithelial sloughing. Epithelial detachment can occur as an artifact of tissue processing. To attempt to distinguish these artifacts from sloughing due to experimental injury, we rated “sloughing” as epithelial detachment that was grossly visible to a blinded rater on low 2.5× magnification.

### 2.6 Permeability Assessment

To detect EB, 10× images of membranous VFs were captured with the AxioImager microscope using the Alexa Fluor 647 filter and 90 ms exposure time. For each experiment, all slides were imaged during a single session within 1 week of sectioning. Before imaging, slides were stored unstained in covered slide boxes. For each mouse, three VF sections 10 μm apart were imaged, quantified, and averaged. EB in select H&E‐stained VF, trachea, lung and esophagus sections was imaged for qualitative assessment of dye location.

EB autofluorescence was quantified by a blinded investigator using ImageJ. Image quantification workflow is presented in Figure S2. VF tissue was selected comprising medial thyroarytenoid muscle and the overlying lamina propria and epithelium. VFs were quantified for percent positive area of fluorescence (% area) and mean fluorescence intensity (MFI). The threshold for positivity was determined using VF images from Experiment 1 to detect a signal in dyed VFs and no signal in mock‐dyed VFs. Subsequently, images of some mock‐dyed VFs from Experiments 2 and 3, and some non‐VF mock‐dyed tissues from Experiment 1, showed evidence of dye transfer through the fixative solution. Since images were taken using the same microscope exposure across all three experiments, we did not adjust the threshold in ImageJ for Experiments 2 or 3. Instead, for each separate experiment (1, 2, and 3), we calculated mean % area and mean MFI of all mock‐dyed VFs in that experiment, then subtracted those average values from the values for each of the dyed VFs [38]. In this way, we controlled for any background signal from the dye in the fixative.

### 2.7 Immunofluorescent Staining

Tissues were stained following standard immunofluorescent protocols. Antigen retrieval was performed by a 20‐min boil in Tris‐based solution, pH 9.0 (Vector H‐3301). Sections were permeabilized for 20 min in 0.1% PBS‐Tween, blocked for 1 h in 5% goat serum, incubated overnight at 4°C in primary antibodies (mouse anti‐p63, Millipore MAB4135, 1:100; rabbit anti‐K14, BioLegend 905301, 1:1000; mouse anti‐ZO‐1, Invitrogen 33‐9100, 1:35; rabbit anti‐laminin, Sigma‐Aldrich L9393, 1:35), and incubated for 1 h at room temperature in secondary antibodies (goat anti‐mouse Alexa Fluor 488, Invitrogen A32723, 1:500 for p63 and 1:200 for ZO‐1; goat anti‐rabbit Alexa Fluor 647, Invitrogen A21244, 1:200). EB autofluorescence was removed with a quenching kit (Vector SP‐8500‐15) following manufacturer instructions. Slides were cover slipped in antifade mounting medium with DAPI (Vector H‐1800) and 20× images were taken. DAPI‐ and p63‐positive nuclei along the length of the VF epithelium, defined by the boundaries of the medial thyroarytenoid muscle, were counted by a blinded investigator using the ImageJ macro “RGB fluorescent cell count v1.32.ijm” developed by Dr. David Ornelles (Wake Forest University) [22, 39]. ZO‐1 and laminin were assessed qualitatively [39].

### 2.8 Statistical Analysis

For presence of sloughing and visible fluorescence, 20% of ratings were randomly repeated. Intra‐rater agreement was 100%. For % area, MFI, number of p63+ cells, and number of DAPI+ cells, 20% of measurements were randomly repeated and intra‐rater reliability was assessed using intraclass correlation coefficients (ICC) based on a single‐rater, absolute‐agreement, two‐way mixed‐effects model [40]. ICCs were excellent (Table S2). Categorical data were tested for group differences using Fisher's exact test. Continuous data were tested for group differences using the Kruskal–Wallis test with Dunn's test for multiple comparisons. The relationship between age and permeability was assessed by simple linear regression. Tests were two‐tailed with *α* = 0.05. Data were analyzed and graphed using GraphPad Prism 10 and SPSS 31.

## 3 Results

### 3.1 Experiment 1: VF Injury and Permeability

#### 3.1.1 Injury

**FIGURE 1:**
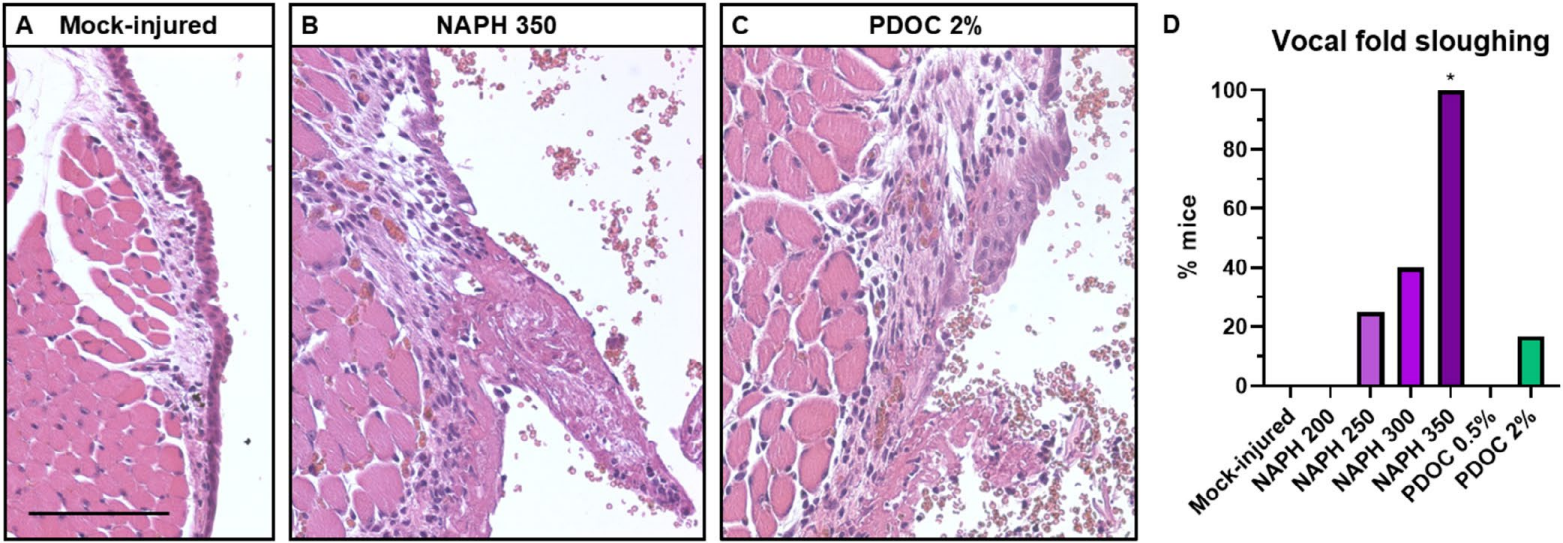
NAPH and PDOC injure mouse VFs. Representative H&E‐stained images of (A) uninjured VF day 1 after mock injury and (B, C) extensive VF epithelial sloughing and fibrin deposition day 1 after 350 mg/kg IP NAPH injection (B) and 10 μL OA instillation with 2% PDOC (C). Scale bar = 100 μm. (D) Prevalence of VF injury. Group numbers: Mock‐injury with 10 μL PBS OA, *n* = 6; NAPH 200, *n* = 4; NAPH 250, *n* = 4; NAPH 300, *n* = 5; NAPH 350, *n* = 7; 10 μL OA 0.5% PDOC, *n* = 5; 10 μL 2% PDOC, *n* = 6. *Significant difference from the mock‐injured group, Fisher's exact test with *p* ≤ Bonferroni‐adjusted *α* = 0.05/6 = 0.0083.

One mouse injured with 2% PDOC (1/7) died within 24 h and was not included in further analyses. Mock injury treatment resulted in no grossly visible VF injury (Figure 1A). VF epithelial sloughing was induced in 100% of mice treated with 350 mg/kg NAPH (Figure 1B,D). For other NAPH doses, sloughing was 20%–40% (Figure 1D). Ten microliters of 2% PDOC resulted in 17% of mice with sloughing (1/6, Figure 1C,D). Injury rate was significantly higher with NAPH 350 than mock injury (*p* = 0.0006, Figure 1D). We observed mild epithelial detachment in other injury groups, but did not quantify this observation because mild detachment could have occurred during histology processing. Our findings show that VFs are injured by NAPH and PDOC in a dose‐dependent manner.

#### 3.1.2 Dye Instillation

**FIGURE 2:**
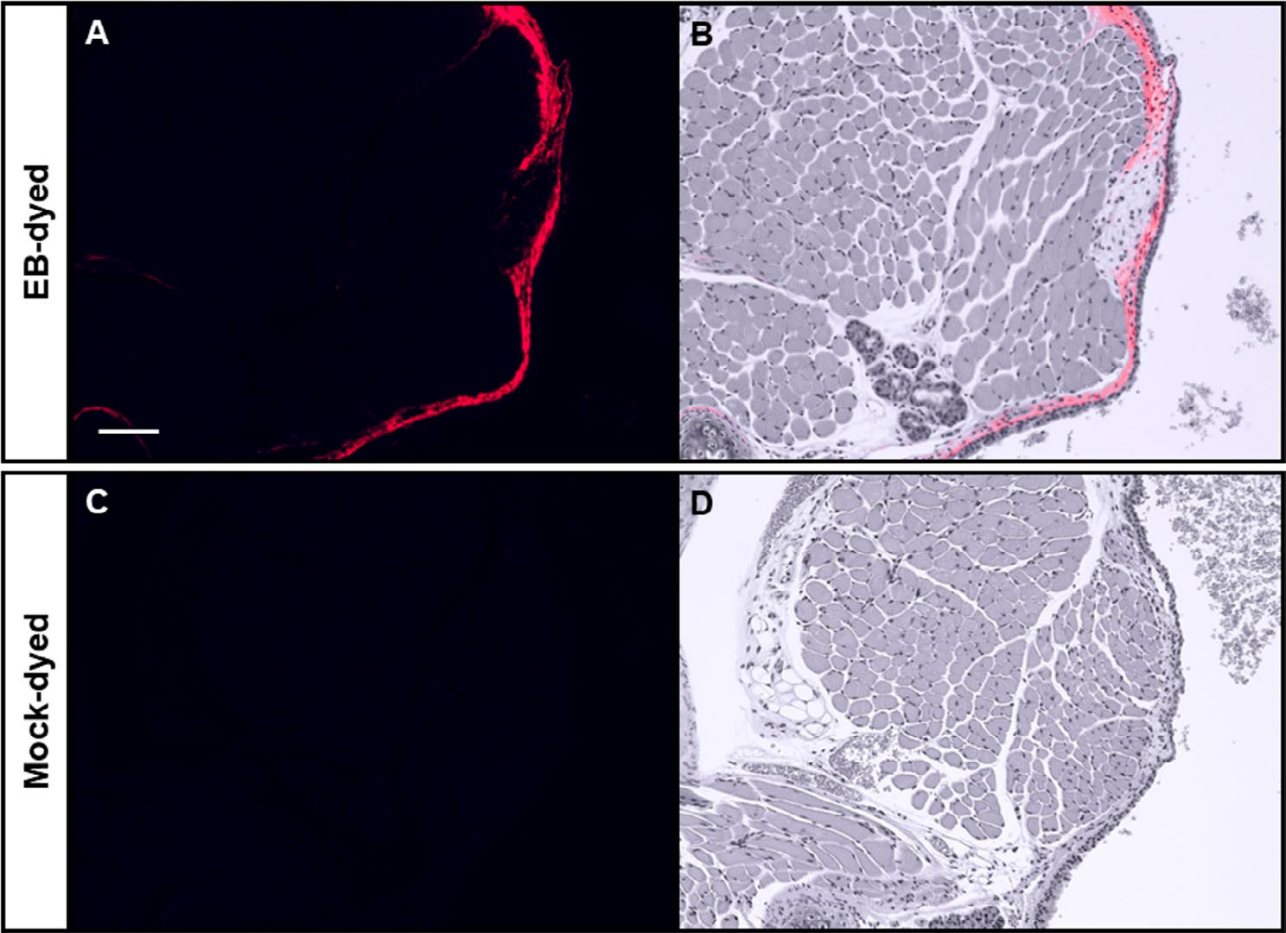
EB penetrates VFs and binds lamina propria. Representative images of uninjured mouse VFs after OA instillation with EB dye (A, B) or mock dye (C, D). H&E‐stained slides were imaged with far‐red filter and 90 ms exposure time (A, C) and with brightfield filter. Fluorescent images were inverted, recolored, and overlaid on brightfield images to localize EB within tissue (B, D). Scale bar = 100 μm.

**FIGURE 3:**
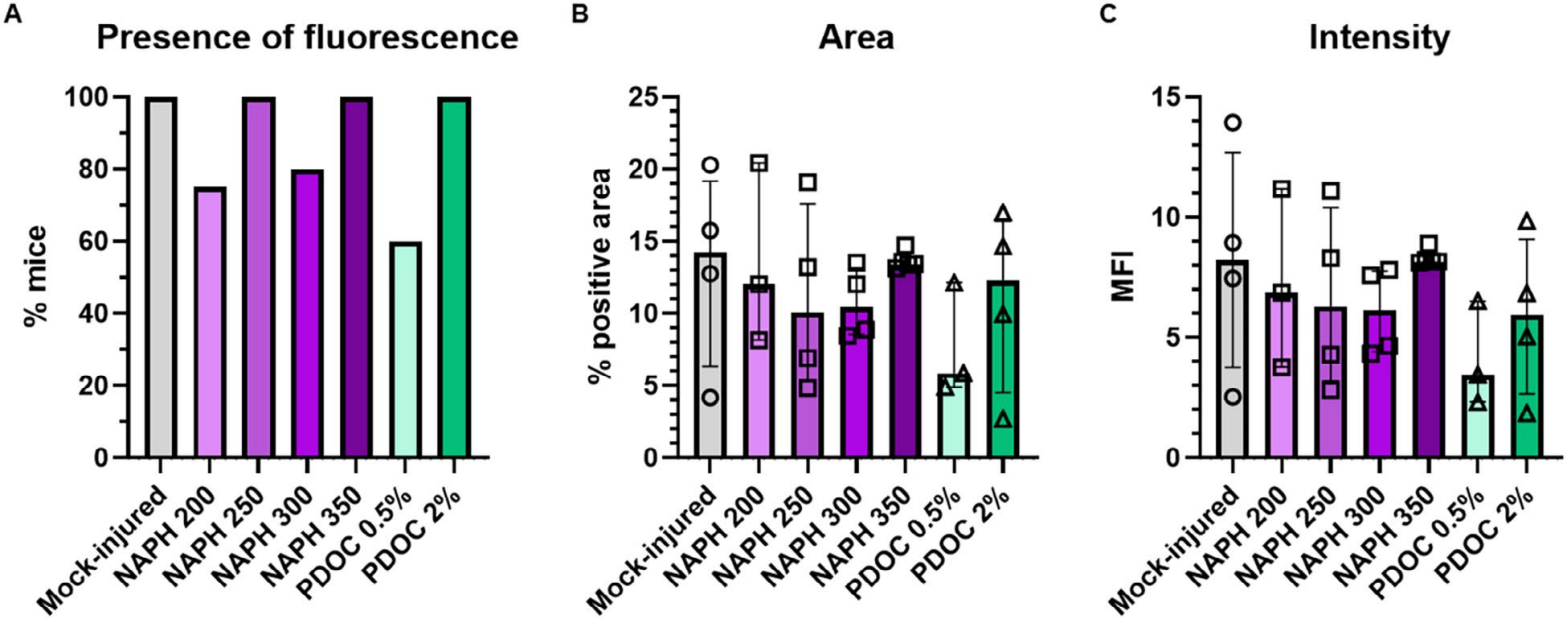
VF injury does not increase permeability to EB dye. (A) Presence of fluorescence in EB‐dyed VFs, *p* > 0.05, Fisher's exact test. Group numbers: Mock‐injured, *n* = 4; NAPH 200, *n* = 4; NAPH 250, *n* = 4; NAPH 300, *n* = 5; NAPH 350, *n* = 5; 10 μL PDOC 0.5%, *n* = 5; 10 μL PDOC 2%, *n* = 4. (B) Area and (C) intensity of EB fluorescence in VFs, *p* > 0.05, Kruskal–Wallis tests. Only EB‐dyed VFs with visible fluorescent signal as indicated in (A) were quantified. Group numbers: Mock‐injured, *n* = 4; NAPH 200, *n* = 3; NAPH 250, *n* = 4; NAPH 300, *n* = 4; NAPH 350, *n* = 5; 10 μL PDOC 0.5%, *n* = 3; 10 μL PDOC 2%, *n* = 4.

We looked for blue dye immediately after dissection, then for red autofluorescence in unstained sections. Blue dye was visible in 84% of larynges (26/31), as well as 84% of tracheas and esophagi and 45% of lungs (14/31). Red autofluorescence was detected in 87% of VFs (27/31; Figure 2A,B). This ranged from 60%–100% by treatment group but did not differ significantly (*p* = 0.55; Figure 3A). There was no dye or autofluorescence in mock‐dyed VFs (Figure 2C,D). These results indicate that 10 μL OA can instill VFs with a variable success rate.

**FIGURE 4:**
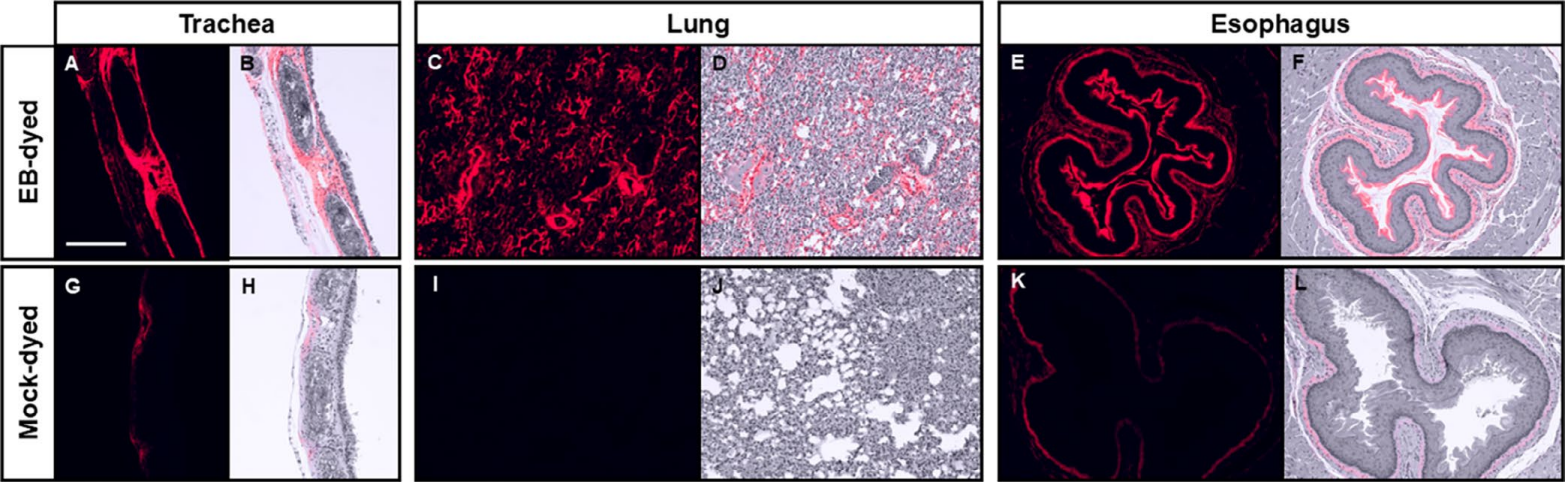
EB localization in trachea, lung, and esophagus. Representative images of mouse tissues after OA instillation with EB dye (A–F) or mock dye (G–L). H&E‐stained slides were imaged with far‐red filter and 90 ms exposure time (A, C, E, G, I, K) and with brightfield filter. Fluorescent images were inverted, recolored, and overlaid on brightfield images to localize EB within tissue (B, D, F, H, J, L). Scale bar = 200 μm.

Red autofluorescence was localized in VF lamina propria (Figure 2), indicating that EB penetrated through the epithelium and bound to collagen and elastin. Dye also bound the lamina propria and other connective tissue in the trachea and lungs (Figure 4A–D) and strongly bound the keratinized layer of mouse esophageal epithelium (Figure 4E,F). Some mock‐dyed mice had weak red autofluorescence in the lateral portions of tracheas and esophagi (Figure 4G,H,K,L), but not in the tracheal or esophageal lumen or lung parenchyma (Figure 4G–J). Because dyed and mock‐dyed tissues were fixed together, this suggests that EB from dyed tissues diffused through the fixative solution into some undyed organs. Therefore, within each experiment, quantitative measurements were normalized to measurements from mock‐dyed tissues.

#### 3.1.3 Permeability

EB‐treated VFs with a visible fluorescent signal were quantified (*n* = 3–5 per group). There was no effect of injury on % area (*p* = 0.61; Figure 3B) or MFI (*p* = 0.39; Figure 3C), indicating that injury did not increase the penetration of EB dye into VF tissues.

### 3.2 Experiment 2: High‐Volume Dye

**FIGURE 5:**
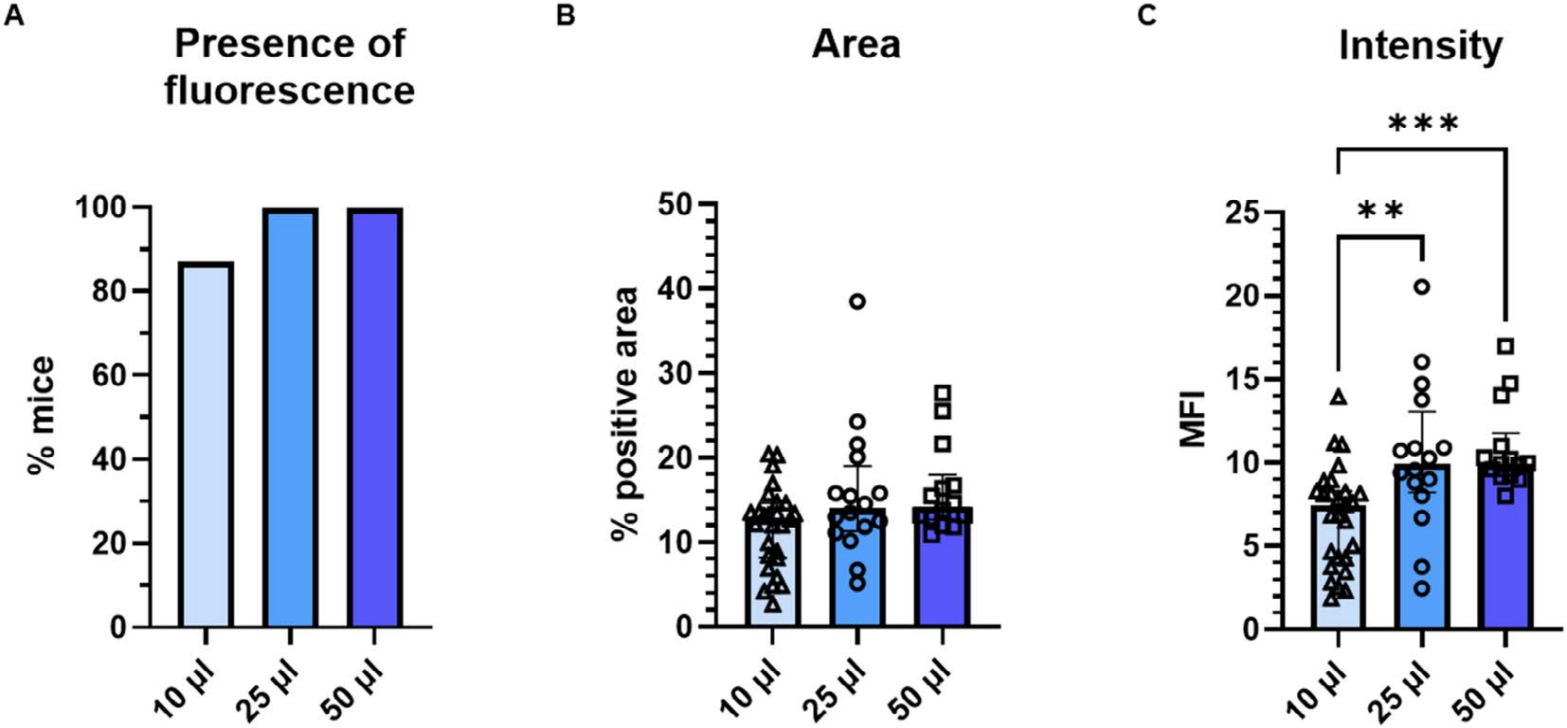
Higher OA volumes increase EB binding in VFs. VFs of mice instilled with 25 and 50 μL EB dye (Experiment 2, *n* = 16 and *n* = 14, respectively) were compared to each other and to mice instilled with 10 μL EB dye in Experiment 1 (A: *n* = 31 instilled; B and C: *n* = 27 with red autofluorescence). (A) Presence of fluorescence in EB‐dyed VFs, *p* > 0.05, Fisher's exact test. (B) Area of EB fluorescence in VFs, *p* > 0.05, Kruskal–Wallis test. (C) Intensity of EB fluorescence in VFs. **Adjusted *p* ≤ 0.01, ***adjusted *p* ≤ 0.001, Dunn's multiple comparisons test after significant Kruskal–Wallis test (*p* = 0.0002).

With instillation of 50 μL EB, dye often spread outside the mouth. The duration required for aspiration pushed the limits of isoflurane anesthesia. Mice instilled with 50 μL EB exhibited moderate dyspnea, hunched posture, and reduced activity. Dye quickly circulated systemically, evidenced by blue‐tinted ears, paws, and tails. With 25 μL EB, dye remained in the oropharynx until aspiration; mice exhibited mild or no dyspnea and typical activity; and only ears turned blue. There were no adverse effects in mock‐dyed mice. Two samples from the 50‐μL group were excluded from permeability assessment, one because the mouse visibly swallowed dye and therefore an unknown volume was aspirated, and the other because adequate VF sections were not obtained. Fluorescence was found in all other VFs instilled with 25 or 50 μL EB (30/30; Figure 5A). Volume did not affect % area (*p* = 0.079, Figure 5B). MFI was higher than 10 μL EB (pooled data from Experiment 1) for both 25 μL (*p* = 0.0055, Figure 5C) and 50 μL (*p* = 0.0003). In summary, higher OA volumes improve VF instillation but increase risks of contamination and morbidity.

### 3.3 Experiment 3: High‐Volume PDOC

**FIGURE 6:**
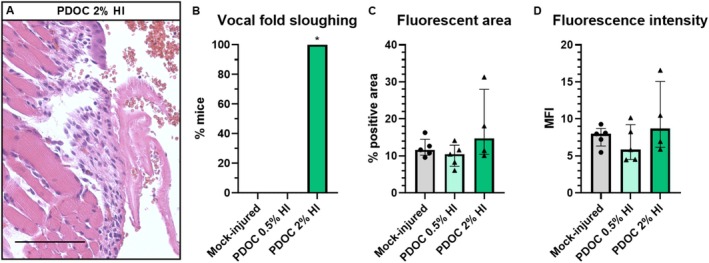
High‐volume PDOC injures mouse VFs but does not increase permeability. (A) Representative H&E‐stained image of extensive VF epithelial sloughing and fibrin deposition day 1 after 25 μL 2% PDOC OA instillation. Scale bar = 100 μm. (B) Prevalence of VF injury. *Significant difference from mock‐injured group, Fisher's exact test with *p* ≤ Bonferroni‐adjusted *α* = 0.05/2 = 0.025. Group numbers including dyed and mock‐dyed mice: Mock‐injury with 25 μL OA PBS, *n* = 7; 25 μL PDOC 0.5%, *n* = 5; 25 μL PDOC 2%, *n* = 6. (C) Area and (D) intensity of EB fluorescence in VFs of EB‐dyed mice, *p* > 0.05, Kruskal–Wallis tests. All EB‐dyed mice had visible fluorescent signal. Group numbers of EB‐dyed mice: Mock‐injured 25 μL PBS, *n* = 5; 25 μL PDOC 0.5%, *n* = 5; 25 μL PDOC 2% *n* = 4.

Two mice in the high‐volume 25 μL 2% PDOC group died within 24 h and were excluded from analyses. VF injury was 100% after high‐volume 2% PDOC (Figure 6A,B) and 0% after high‐volume 0.5% PDOC and mock injury. Injury was greater with high‐volume 2% PDOC than with high‐volume mock injury (*p* = 0.0006; Figure 6B). All EB‐treated VFs (14/14) autofluoresced. There were no group differences in % area (*p* = 0.35, Figure 6C) or MFI (*p* = 0.44, Figure 6D). This experiment showed that high‐volume 2% PDOC reliably injures VFs but causes relatively high mortality and confirmed that injury does not increase VF permeability in mice.

### 3.4 Age and Sex Differences

We assessed the effects of age and sex on VF sloughing and permeability measures (% area and MFI). There was no age difference between mice with and without VF sloughing after injury treatments (Figure S3A). In mice that received EB dye, age was associated with permeability in linear regression, with small but statistically significant slopes (% positive area: *β* = 0.64, *p* = 0.017; MFI: *β* = 0.60, *p* = 0.0002; Figure S3B). With regard to sex, more females than males exhibited VF sloughing, but this difference was not statistically significant (Figure S4A). Permeability measures were higher in females than in males (*p* = 0.023 and 0.024, Figure S4B,C). However, total VF area was larger in males than in females (*p* = 0.031, Figure S4D). This means that it is not clear whether the sex differences in % area and MFI are a result of true differences in permeability, or an artifact of proportionally smaller medial thyroarytenoid muscles relative to lamina propria in female mice, which was the tissue layer where dye was found. Age was not associated with total VF area (Figure S3C).

### 3.5 Injury Characterization

**FIGURE 7:**
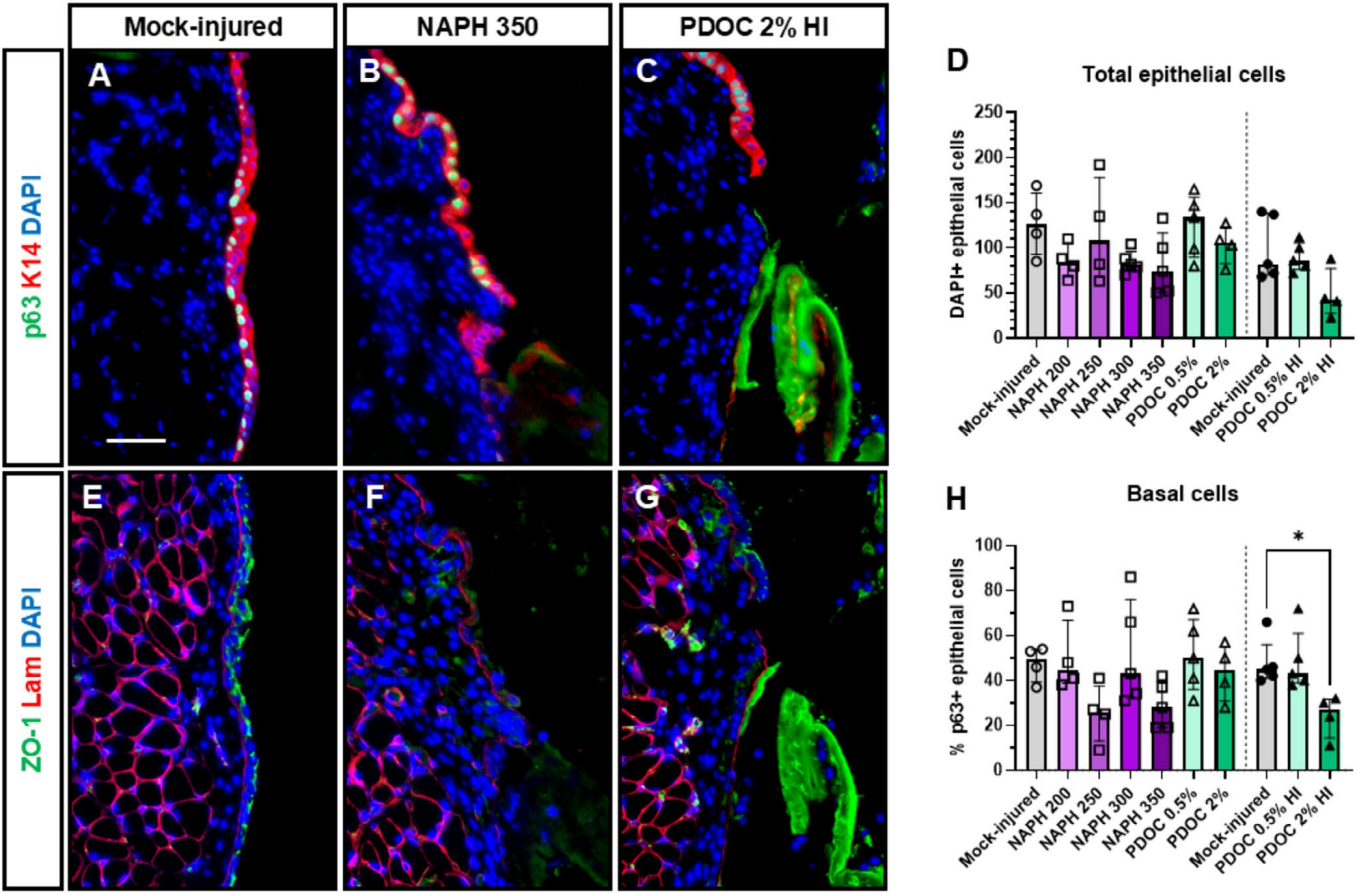
Basal cells, tight junctions, and basement membrane in mouse VFs day 1 post injury with NAPH and PDOC. Representative immunofluorescent images of uninjured VF day 1 after mock injury (A, E), and injured VF day 1 after 350 mg/kg IP NAPH injection (B, F) or 25 μL 2% PDOC OA instillation (C, G). Top row (A–C): p63 (green) and K14 (red) costain, DAPI nuclear counterstain (blue). Bottom row (E–G): ZO‐1 (green) and laminin (red) costain, DAPI nuclear counterstain (blue). Scale bar = 50 μm. (D) Total epithelial cells quantified as number of DAPI+ epithelial cells per image, *p* > 0.05, Kruskal–Wallis tests. Experiment 1 and Experiment 3 data were statistically analyzed separately. (H) Basal epithelial cells quantified as percent of DAPI+ epithelial cells that were p63+. *Adjusted *p* < 0.05, Dunn's multiple comparisons test after significant Kruskal–Wallis test (*p* = 0.0082).

To determine the effect of NAPH and PDOC on VF epithelium day 1 post injury, we stained for p63 (basal cells), ZO‐1 (tight junctions), and laminin (basement membrane). Areas of extensive sloughing induced by both chemicals were p63‐negative (Figure 7A–C). There were fewer total epithelial cells in groups with more extensive injury, but this was not statistically significant (Figure 7D). Basal cells within the remaining epithelium were decreased compared to mock‐injured controls, which was statistically significant for high‐volume 2% PDOC (*p* = 0.020, Figure 7H). We did not attempt to quantify cytoplasmic, membranous, or extracellular immunofluorescent stains due to nonspecific binding of secondary antibodies to sloughing tissue. Based on qualitative observations, tight junction marker ZO‐1 was decreased in injured VFs (Figure 7E–G). Basement membrane was largely intact, forming a continuous line above the lamina propria (Figure 7F,G). However, there were some discontinuous areas that could be attributed to in vivo injury or sectioning artifact.

## 4 Discussion

We have described low‐tech, low‐cost, rapid methods to reliably injure and instill mouse VFs. IP injection and OA instillation use common laboratory supplies, do not involve endoscopy or microscopy, require brief or no anesthesia, and can be performed by a single investigator in < 30 s per mouse. NAPH and PDOC are relatively common, inexpensive chemicals that injure murine VF epithelium in a dose‐dependent manner. Using EB dye, we demonstrated that OA instills murine VFs with liquid solutions.

NAPH at 350 mg/kg induced sloughing in 100% of mouse VFs. Our results are consistent with published dose effects of IP NAPH. Others have shown that 50 mg/kg injures lungs [41], 250 mg/kg injures trachea [42], and 275 mg/kg induces hyperplasia in 60% of VFs [22]. Topical PDOC‐induced injury is also dose‐dependent. The standard dose for tracheal injury is 2% in 10–15 μL [8, 10, 12]. In nasal respiratory epithelium, 1% PDOC causes visible damage, while 0.1% increases permeability without apparent injury [26]. In nasal stratified squamous epithelium, 1% is insufficient for injury [26]. We found that 2% PDOC instilled via OA injures murine VFs, with 100% success in a 25‐μL volume. Our 85%–90% survival rate after 2% PDOC was consistent with the literature [27]. We did not collect tissues of mice that died before the EB permeability test, but 25 μL of 2% PDOC delivered intratracheally can induce lung hemorrhages [27]. In our experiments, 350 mg/kg NAPH best balanced reliable VF injury with survival. However, because this dose is very close to the median lethal dose of 380 mg/kg [43], mortality is expected with higher animal numbers or longer experiments. Administering NAPH via inhalation is one strategy to overcome this limitation. Inhaled NAPH has the potential to increase VF injury while avoiding severe lung injury because this delivery method induces greater injury in proximal airways than distal airways [44].

The VF injuries induced by NAPH and PDOC are characterized by epithelial sloughing. This is unlike surgical injury, which is the type of injury that is most often used to study VF wound healing. However, epithelial surface damage, discontinuity, and/or desquamation have been described in animal models of acute phonotrauma [45, 46, 47, 48, 49]. Experimental human studies of phonotrauma with histological outcomes cannot be done. It is possible that the mild sloughing induced by low doses of NAPH and PDOC could be analogous to phonotraumatic injury. Further investigation is required to understand potential parallels between mild chemical vocal fold injury and phonotraumatic vocal fold injury, but the existence of such parallels would be of high translational relevance to human voice production. To conduct such studies, improved quantitative measures of injury are required, particularly to distinguish mild epithelial injury from tissue processing artifacts. Longer experiments in the mouse model are warranted to understand VF wound healing after NAPH and PDOC injury, and to understand changes in VF permeability during acute wound healing and chronic tissue remodeling.

The mechanism of NAPH injury in VFs by club cell ablation has been validated [22]. Thus, we focused on validating the mechanism of PDOC injury in VFs by staining for basal cells and tight junctions. We characterized the effects of PDOC (and NAPH) on VF basal cells because, in respiratory epithelium, there are conflicting reports as to whether PDOC leaves basal cells intact while removing suprabasal cells [8, 9, 26]. If this were the case, basal cells as a percentage of epithelial cells would increase after injury. However, percent p63+ basal cells in injured VFs did not change in most injury groups, and decreased in the case of 25 μL of 2% PDOC. Therefore, PDOC injury in VFs did not leave basal cells intact. We stained for ZO‐1 because it has been stated that PDOC disrupts tight junctions [9, 26]. We hypothesized that we might see subtle changes in ZO‐1 at lower doses of PDOC or NAPH. There was a qualitative decrease in ZO‐1 after injury, but we ultimately did not attempt to quantify ZO‐1 due to nonspecific binding of secondary antibodies to sloughing tissue. Decreased basal cells and tight junctions can be explained as a natural consequence of epithelial loss in VF injury. Intact basement membrane indicates that chemical VF injury targets damage to the epithelium more precisely than surgical VF injury in mice. Assessing later timepoints after injury, or using alternative approaches like transmission electron microscopy or Western blotting, could allow for quantification of tight junctions and basement membrane.

We demonstrated that 10 μL OA can instill VFs in 100% of mice in a given experiment but has potential for variability. Instillation is virtually guaranteed with 25–50 μL. Up to 50 μL OA is a typical volume for lung instillation [34]. We confirmed that 50 μL PBS is safe, but found high morbidity with 50 μL EB. Others have shown acceptable toxicity of ~50 μL of various solvents delivered to mice via OA [50]. Oral containment of 50 μL was challenging, which would contraindicate inoculation with pathogens. Therefore, we recommend VF instillation with 10–25 μL. If higher volumes are needed, preliminary experiments are indicated to determine the safety of a given compound.

EB dye (961 Da) penetrates epithelium and binds lamina propria in uninjured mouse VFs. The amount of bound EB does not increase with injury. This is unlike VF and laryngeal epithelial permeability tested ex vivo. In vivo gastric reflux increases (but is not required for) ex vivo murine anterior commissure permeability to sodium fluorescein (376 Da) [31, 35, 51, 52]. In vivo surgical injury is required for permeability of ex vivo rat VFs to sulfo‐N‐hydroxysuccinimide (NHS)‐long chain (LC)‐biotin (556 Da) [53] and lanthanum nitrate [54] (324 Da [55]). Ex vivo histamine increases ex vivo sheep VF permeability to horseradish peroxidase (HRP; 40 kDa) [56]. To our knowledge, this is the first in vivo study of VF permeability, so our results might not be comparable to ex vivo experiments. Consistent with our findings, uninjured ex vivo mouse anterior commissure is permeable to fluorescein [31, 35, 51, 52]. Our in vivo papillomavirus infection studies [5, 32] suggest that intact mouse laryngeal and respiratory epithelium can be penetrated by papillomavirus (60 nm [57]; ~20 MDa). Therefore, mouse VF epithelium is highly permeable at baseline. This is consistent with TEER measures of 30–50 Ω*cm^2^ for mouse anterior commissure [31, 35, 51, 52], well below the accepted threshold of 300 for “tight” epithelium [58].

Larger or differently charged molecules might affect VF permeability. We detected EB in lamina propria and minimally in epithelium, indicating that dye penetrated epithelium through the paracellular pathway. Paracellular spaces in airway and VF epithelia are sensitive to ionic charge [59, 60]. Comparing ionic charges of the compounds used to test VF permeability, EB, fluorescein, and sulfo‐NHS‐LC‐biotin are negatively charged in solution [61, 62, 63], while lanthanum is positively charged [55] and HRP has been described as neutral [56]. Papillomavirus capsids can be positively or negatively charged, depending on viral genus [64], but the charge of the mouse papillomavirus specifically is unknown. EB can be conjugated to serum albumin, and this compound is much larger than unbound dye (69 kDa [65] vs. 961 Da) and maintains a negative charge [66]. Future studies could employ this and other molecules to fully explore the effect of molecular size and charge on VF permeability in vivo. Given that we found minor age and sex differences in permeability, these characteristics should be controlled for and further explored.

The high permeability of mouse VFs should not prevent the use of mice as a model species for laryngology research. VF permeability and barrier vary between species and within species. Human VF TEER and permeability are unknown in vivo and ex vivo. VFs of pigs [67] and sheep [68] exceed the TEER threshold for “tight” epithelium, while VFs of rabbits [69] and dogs [68] do not. Human VF epithelium grown in 3D organotypic culture in vitro has been described as “immature” with altered barrier proteins and reduced barrier function [70, 71]. Therefore, high permeability is a common feature of tractable preclinical VF model systems. Regardless, the larynges of humans, mice, and other species have abundant squamociliary junctions where agents can potentially enter epithelium [72, 73]. As evidence that mouse models are imperfect but useful, both pigs (“tight” VF epithelium) and mice (“leaky” VF epithelium) have been used to show compromised barrier with exposure to gastric reflux, and mice have further been used to demonstrate that anti‐reflux agents have a protective effect on laryngeal epithelial permeability [31, 35, 51, 52, 67].

The primary limitations of our murine VF injury and instillation methods are off‐target effects. NAPH and PDOC also injure respiratory tissues at the doses required to affect VFs. Surgical VF injury has off‐target effects as well, including inadvertent injury of bilateral VFs, supraglottis, pharynx, and/or tongue [5, 6]. There were also off‐target effects of OA VF instillation. Consistent with the literature [74], OA delivered dye to both respiratory and digestive tracts. However, high‐volume instillation delivered a maximal dose of EB to VFs. Future studies could determine the precise dose delivered to VFs using careful dissection and spectrophotometry for EB fluorescence [65]. Investigators must choose which off‐target effects are acceptable when planning murine VF procedures.

The injury and instillation methods we have described have a number of translational applications. One such application is furthering basic understanding of laryngeal biology. As an example, NAPH and PDOC have been used to wound the mouse trachea for studies of tissue organization and stem cell biology during regeneration, with parallel experiments in human cells and tissues that validate major findings [10]. Similar basic work can now be done in the VFs. The ability to increase or decrease the extent of expected VF injury by varying the dose of a chemical reagent provides the opportunity for nuanced studies of injury “dose.” The ability to deliver reagents into mouse VF tissue has a vast array of translational applications. Perhaps the most exciting of these is the ability to spatially target genetic engineering approaches to the VFs by instilling reagents that induce gene expression or excision in transgenic mice.

## 5 Conclusion

We have successfully repurposed low‐tech, quick, and reliable injury and instillation methods established in respiratory research to advance preclinical VF research. Two chemicals, NAPH and PDOC, induce mouse VF epithelial sloughing in a dose‐dependent manner. OA instills mouse VFs in a volume‐dependent manner. Using these methods, we showed that uninjured murine VF epithelium is highly permeable in vivo, and that permeability does not increase with injury. Inherent permeability of mouse VFs will facilitate targeted genetic engineering approaches and studies of hazards and drug treatments, but findings should be supported by cross‐species validation in light of species differences in VF permeability.

## Conflicts of Interest

The authors declare no conflicts of interest.

## Supporting information


**Data S1:** Supporting Information.

## Data Availability

The data that support the findings of this study are openly available in Dryad at http://doi.org/10.5061/dryad.9w0vt4bsc.
