# Supplementary material for: Mouse Vocal Fold Permeability In Vivo: Effects of Novel Low‐Tech Injury and Instillation Methods
Source: Laryngoscope. 2025 Dec 10;136(4):1846–57. doi: 10.1002/lary.70266 (PMC12993102; doi:10.1002/lary.70266)
Supplement: Supplementary file 1 — Data S1: Supporting Information. [file LARY-136-1846-s001.docx]

**SUPPORTING INFORMATION**

**Mouse vocal fold permeability *in vivo*: Effects of novel low-tech injury and instillation methods**

**Running Title:** Mouse vocal fold injury and permeability

**Authors**: Renee E. King, PhD; Ella T. Ward-Shaw, BS; and Paul F. Lambert, PhD

**Affiliation**: McArdle Laboratory for Cancer Research, Department of Oncology, University of Wisconsin-Madison, Madison, WI

**Corresponding Author**:

Renee E. King

McArdle Laboratory for Cancer Research

1111 Highland Ave, WIMR 6459M

Madison WI 53705, USA

Tel: 608-262-6407

Email: [renee.king@wisc.edu](mailto:renee.king@wisc.edu)

**Table S1. Animal numbers for each experimental group.**

| **Experiment 1: VF Injury and Permeability** | | | | |
| --- | --- | --- | --- | --- |
| **Injury** | **Dye** | **Males** | **Females** | **Total N** |
| PBS OA 10 μl (mock) | PBS OA 10 μl (mock) | 1 | 1 | 2 |
|  | EB OA 10 μl | 3 | 1 | 4 |
| NAPH 200 mg/kg IP | EB OA 10 μl | 3 | 1 | 4 |
| NAPH 250 mg/kg IP | EB OA 10 μl | 3 | 1 | 4 |
| NAPH 300 mg/kg IP | EB OA 10 μl | 4 | 1 | 5 |
| NAPH 350 mg/kg IP | PBS OA 10 μl (mock) | 1 | 1 | 2 |
|  | EB OA 10 μl | 3 | 2 | 5 |
| 0.5% PDOC OA 10 μl | EB OA 10 μl | 4 | 1 | 5 |
| 2.0% PDOC OA 10 μl | PBS OA 10 μl (mock) | 1 | 1 | 2 |
|  | EB OA 10 μl | 3 | 1 | 4 |
|  | None^a^ | 0 | 1 | 1 |
| **Experiment 2: High-Volume Dye** | | | | |
| **Injury** | **Dye** | **Males** | **Females** | **Total N** |
| None | PBS OA 25 μl (mock) | 2 | 1 | 3 |
|  | PBS OA 50 μl (mock) | 3 | 0 | 3 |
|  | EB OA 25 μl | 8 | 8 | 16 |
|  | EB OA 50 μl | 7 | 8 | 15 |
|  | EB, unknown volume^b^ | 1 | 0 | 1 |
| **Experiment 3: High-Volume PDOC** | | | | |
| **Injury** | **Dye** | **Males** | **Females** | **Total N** |
| PBS OA 25 μl (mock) | PBS OA 10 μl (mock) | 0 | 2 | 2 |
|  | EB OA 10 μl | 2 | 3 | 5 |
| 0.5% PDOC OA 25 μl | EB OA 10 μl | 3 | 2 | 5 |
| 2.0% PDOC OA 25 μl | PBS OA 10 μl (mock) | 0 | 2 | 2 |
|  | EB OA 10 μl | 2 | 2 | 4 |
|  | None^a^ | 1 | 1 | 2 |

a: Mice died before 24 hours and did not receive dye. Tissues were not assessed.

b: Mouse swallowed during OA procedure and the aspirated dye volume could not be determined. Tissues were not assessed.

VF: vocal fold. PBS: phosphate-buffered saline. EB: Evans blue dye. OA: oropharyngeal aspiration. NAPH: naphthalene. PDOC: polidocanol.


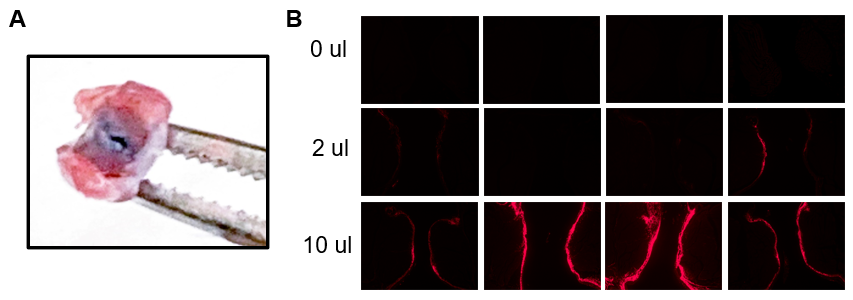


**Figure S1. Oropharyngeal aspiration with a 10-μl volume reliably instills mouse vocal folds.** FVB/NTac mice (n = 4 per group) were instilled with 0, 2, or 10 μl of 5% Evans blue dye and sacrificed after 30 minutes. (A) Visible blue dye within a dissected larynx 30 minutes after 10 μl instillation. (B) Fluorescent microscopy of unstained formalin fixed, paraffin embedded slides showing Evans blue dye autofluorescence in coronal sections of the vocal folds. Slides were imaged with an Alexa 647 filter using consistent exposure times.


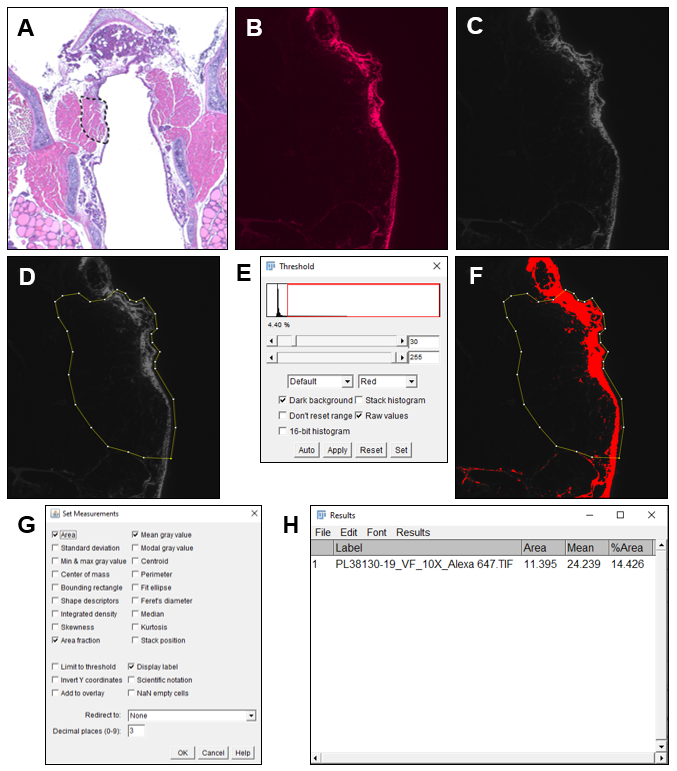
**Figure S2. Workflow for vocal fold permeability quantification.**

(A) Low-magnification H&E-stained coronal section of mouse larynx to illustrate the quantified area of the vocal fold, or region of interest (ROI). Dashed line encompasses the medial thyroarytenoid muscle, lamina propria, and epithelium.

(B) High-magnification image of mouse vocal fold in the unstained slide adjacent to (A), depicting red autofluorescence of Evans blue dye.

(C) Image in (B) after conversion to grayscale in ImageJ (command: Image > Type >8-bit).

(D) Image in (C) after selecting the ROI (Polygon tool).

(E) Threshold window (Image > Adjust > Threshold) depicting the threshold used for all experiments (30-255, dark background).

(F) Image in (D) after thresholding. Red pixels are over the threshold.

(G) Measurement settings window (Analyze > Set Measurements) depicting measurements collected for all experiments.

- “Area”: total ROI.
- “Area fraction”: percent of pixels in the ROI that are over the threshold; program output reads “%Area”; presented as “% area” and “% positive area” in this study.
- “Mean gray value”: mean intensity of all pixels in the ROI, whether or not they are over the threshold; program output reads “Mean”; presented as “mean fluorescence intensity (MFI)” in this study.

(H) Measurement results window after the ROI is measured (Analyze > Measure).

**Table S2. Intra-rater reliability of quantitative imaging measurements.**

| **Measurement** | **Total N quantified^a^** | **N repeated^b^** | **ICC** | **95% CI** | **Interpretation** |
| --- | --- | --- | --- | --- | --- |
| Raw % area | 87 | 18 | 0.97 | 0.91-0.99 | Excellent |
| Raw MFI | 87 | 18 | 0.98 | 0.94-0.99 | Excellent |
| # p63+ epithelial cells | 45 | 9 | 0.93 | 0.75-0.98 | Excellent |
| # total epithelial cells | 45 | 9 | 0.92 | 0.35-0.98 | Excellent |

a: Total number of images quantified for each measurement in Experiments 1-3.

b: Images quantified a second time, comprising 20% of the total images quantified for that measurement.

ICC: intraclass correlation coefficients. CI: confidence interval. MFI: mean fluorescence intensity.


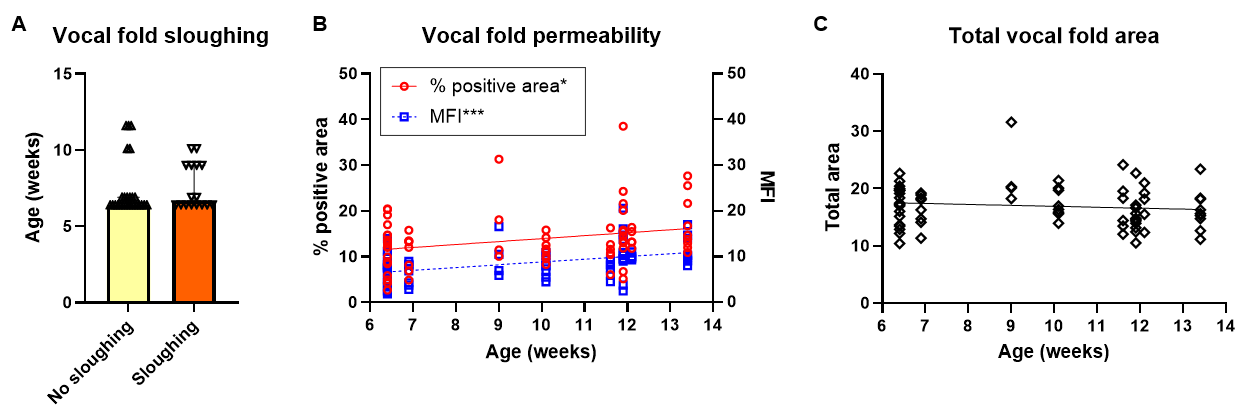


**Figure S3. Age differences in vocal fold injury and permeability.** (A) Age did not differ between mice with no vocal fold sloughing (n = 13, median age = 6.4 weeks) and with vocal fold sloughing (n = 29, median age = 6.7 weeks) after injury treatment. All animals treated with NAPH or PDOC in Experiments 1 and 3 were analyzed (total n = 42). p > .05, Mann-Whitney U test. (B) Permeability measures (% positive area of Evans blue autofluorescence and mean fluorescence intensity [MFI]) were positively associated with age in simple linear regression models with age as the independent variable. All animals treated with any volume of Evans blue dye in Experiments 1, 2, and 3 were analyzed (n = 71). % positive area: β = 0.64, *p = .017). MFI: β = 0.60, ***p = .0002). (C) Total vocal fold area was not associated with age in simple linear regression with age as the independent variable (β = -0.16, p = .32). All animals treated with any volume of Evans blue dye in Experiments 1, 2, and 3 were analyzed (n = 71).


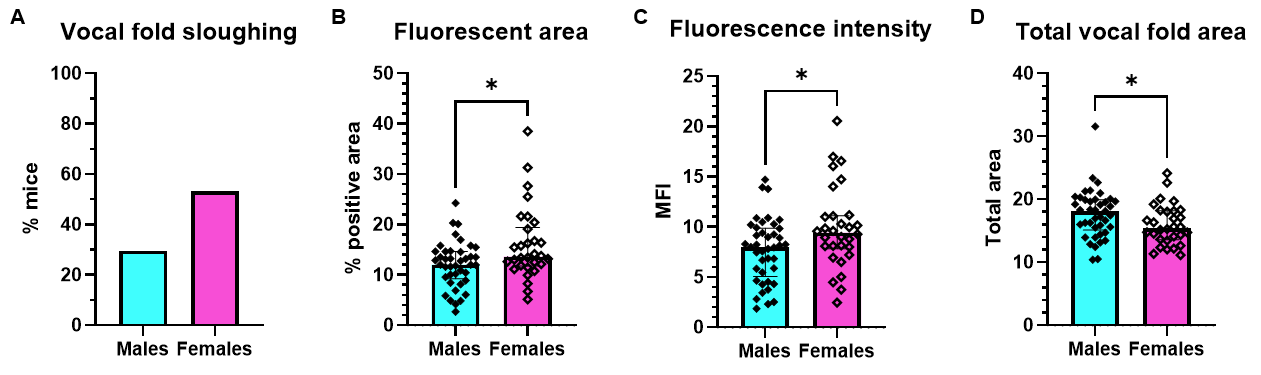


**Figure S4. Sex differences in vocal fold injury and permeability.** (A) Percent of mice with vocal fold sloughing after injury treatment was compared between males (n = 27) and females (n = 15). All animals treated with NAPH or PDOC in Experiments 1 and 3 were analyzed (total n = 42). p > .05, Fisher’s exact test. (B-D) Permeability measures (% positive area of Evans blue autofluorescence and mean fluorescence intensity [MFI]) and vocal fold area (medial thyroarytenoid muscle and overlying lamina propria and epithelium) were compared between males (n = 41) and females (n = 30). All animals treated with any volume of Evans blue dye in Experiments 1, 2, and 3 were analyzed (total n = 71). (B) % positive area, *p = .023, Mann-Whitney U test. (C) MFI, *p = .024, Mann-Whitney U test. (D) Total vocal fold area, *p = .031, Mann-Whitney U test.
